# The effects of glucagon-like peptide-1 receptor agonists on adipose tissues in patients with type 2 diabetes: A meta-analysis of randomised controlled trials

**DOI:** 10.1371/journal.pone.0270899

**Published:** 2022-07-07

**Authors:** Fupeng Liu, Qing Yang, Hongli Zhang, Yanhong Zhang, Guangzhi Yang, Bo Ban, Yanying Li, Mei Zhang

**Affiliations:** 1 Department of Endocrinology, Affiliated Hospital of Jining Medical University, Jining Medical University, Jining, China; 2 Chinese Research Center for Behavior Medicine in Growth and Development, Jining, China; 3 Department of Nutrition, Affiliated Hospital of Jining Medical University, Jining Medical University, Jining, China; Weill Cornell Medical College Qatar, QATAR

## Abstract

**Aims:**

Glucagon‑like peptide 1 receptor agonist (GLP-1RA) treatment can improve adipose distribution. We performed this meta-analysis to investigate whether GLP-1RAs preferentially reduce visceral adipose tissue (VAT) over subcutaneous adipose tissue (SAT) in patients with type 2 diabetes.

**Materials and methods:**

We searched MEDLINE and the Cochrane Library for randomised controlled trials explicitly reporting changes in VAT and SAT. A random-effects model was performed to estimate the weighted mean difference (MD) for VAT and SAT. Heterogeneity among the studies was assessed using *I*^2^ statistics, and publication bias was assessed using Egger’s tests. Meta-regression was performed to identify the correlation between changes in adipose tissues and changes in body weight and glycated haemoglobin level.

**Results:**

Ten trials with 924 patients were enrolled in the meta-analysis. GLP-1RA treatment led to similar absolute area (cm^2^) reductions in VAT (MD -21.13 cm^2^, 95% CI [-29.82, -12.44]) and SAT (MD -22.89 cm^2^, 95% CI [-29.83, -15.95]). No significant publication bias was detected, and this result was stable in the sensitivity and subgroup analyses. Moreover, GLP-1RA treatment resulted in a greater reduction in VAT and SAT in the subgroup with a greater reduction in body weight. The absolute area reduction in VAT was significantly correlated with the reduction in body weight (r = 6.324, p = 0.035).

**Conclusions:**

GLP-1RA treatment leads to significant and similar absolute reductions in VAT and SAT, and the reduction in adipose tissues may be correlated with the reduction in body weight.

## 1. Introduction

Compared with individuals without diabetes mellitus, patients with diabetes have a 2-fold increased risk of vascular diseases and a 2.32-fold increased risk of death from vascular causes [1, 2]. Studies have shown that a high percentage of visceral adipose tissue (VAT) is consistently associated with type 2 diabetes, and obese patients with type 2 diabetes show a greater propensity for ectopic and visceral fat deposition [3, 4]. Compared with subcutaneous adipose tissue (SAT), excessive VAT is more pathogenic and increases the risk of cardiovascular disease, including insulin resistance, hypertension, dyslipidaemia, and systemic chronic low-grade inflammation [5, 6]. Consequently, VAT reduction leads to greater improvement in insulin sensitivity and lower cardiometabolic risk than SAT reduction [7].

Glucagon‑like peptide 1 receptor agonists (GLP-1RAs) are widely used to treat type 2 diabetes. GLP-1RA treatment is associated with improvements in glucose control and reduced cardiovascular events and mortality in patients with type 2 diabetes [8, 9]. Although the exact mechanism is not fully elucidated, GLP-1RAs may exert their cardiovascular protective effects by improving cardiovascular risk factors; for example, GLP-1RA treatment can lead to weight loss, blood pressure reduction, improved lipid profiles and direct effects on the heart and vascular endothelium [10]. Recently, several clinical trials have indicated that GLP-1RA treatment can improve adipose distribution and has advantages in VAT reduction. In addition, immunofluorescence results have confirmed that the GLP-1R protein is present and more abundant in VAT than in SAT in individuals with type 2 diabetes [11]. Therefore, GLP-1RA effects may be specific to VAT. The aim of this study was to conduct a meta-analysis of randomised controlled trials (RCTs) to investigate whether GLP-1RAs preferentially reduce VAT over SAT in patients with type 2 diabetes.

## 2. Methods

This systematic review was performed in accordance with the Preferred Reporting Items for Systematic Reviews and Meta-analyses guidelines (S1 Table) [12]. The primary protocol was registered in PROSPERO (registration number: CRD42021264743). Two authors independently performed literature searches, study data extraction, and risk-of-bias assessments. In the case of disagreement between the two reviewers, consensus was reached through a re-evaluation of the article and consultation with a third author. All analyses in this research were based on previously published studies; thus, no ethical approval or patient consent was needed.

### 2.1 Data sources and searches

We searched the electronic databases MEDLINE (via PubMed) and the Cochrane Library for RCTs or clinical trials from inception to May 1, 2021, using terms including “(‘glucagon-like peptide 1’ OR ‘GLP-1’) AND (‘adipose’ OR ‘fat’)” AND ‘type 2 diabetes’. Additionally, relevant systematic reviews and meta-analyses were searched using these keywords. We consulted content experts and manually screened the references from identified systematic reviews to identify studies missed by our search strategy.

### 2.2 Study selection

We included RCTs that compared GLP-1RAs (with treatment dose included; e.g., liraglutide ≥ 0.9 mg/day) with placebo or standard care in patients with type 2 diabetes. Requirements were a follow-up duration of at least 6 months or 24 weeks and explicit reporting of the absolute changes in VAT and SAT in units of square centimetres. This is an appropriate duration for GLP-1RAs to achieve the majority reduction in body weight and therefore may also reduce adipose tissues the most [13, 14].

### 2.3 Data extraction

The following information from each eligible trial was collected: (1) general study characteristics, including the name of the first author, year of publication, study design, interventions, and duration of follow-up; (2) patient characteristics, including the number of patients randomised, body mass index (BMI), body weight, and the level of glycated haemoglobin (HbA1c); and (3) the absolute changes in VAT and SAT in units of square centimetres. The changes in body weight and the HbA1c level served as a reference.

### 2.4 Risk-of-bias and evidence-quality assessment

Risk of bias was assessed using the Cochrane risk-of-bias tool based on the following criteria: random sequence generation, allocation concealment, the blinding of participants and personnel, the blinding of outcome assessment, the completeness of outcome data, the selectivity of reporting, and other bias [15]. Judgements were expressed as “low risk,” “high risk,” or “unclear risk.”

### 2.5 Data synthesis and analysis

Considering the heterogeneity among the enrolled studies, such as the background of patients, the instrument of measurement, and the type of GLP-1RAs, we employed the inverse-variance method and a random-effects model to pool the outcome data. The pooled results are reported as the weighted mean difference (MD) with the associated 95% confidence interval (CI) using the standard DerSimonian–Laird method. The pooled standard deviation (SD) for the change in all outcomes was imputed (when not available) assuming the correlation coefficient with a conservative value of 0.50 between the baseline and final measurements. We examined heterogeneity among the studies using *I*^2^ statistics with the associated 95% CI [16]. Sensitivity and subgroup analyses were performed according to the time of publication, whether the SD for the change in adipose tissue was available, the types of GLP-1RAs, the baseline BMI and HbA1c level, and the MD in body weight and HbA1c level. We assessed funnel plot asymmetry using Egger’s tests and defined significant publication bias as p < 0.1. The trim-and-fill computation method was used to estimate the effect of publication bias on the interpretation of the results when publication bias was significant [17]. We also performed meta-regression to identify the correlation between the mean change in adipose tissue and the mean change in body weight and HbA1c level. We performed all analyses using RevMan software (version 5.1, Nordic Cochrane Centre; Copenhagen, Denmark) and Stata (version 11, Stata-Corp LP, College Station, TX, USA).

## 3. Results

### 3.1 Study selection

**Fig 1:**
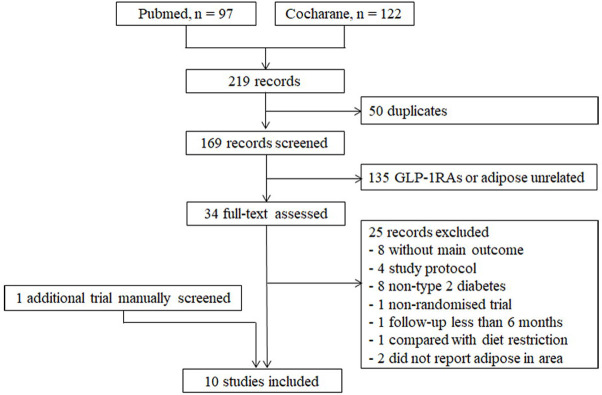
Study flow diagram.

Fig 1 shows the study selection process. A total of 219 studies were identified through the search strategy, and 9 trials were included in this analysis [18–26]. One additional trial meeting the inclusion criteria was identified through manual searching [27].

### 3.2 Characteristics of the included studies

The characteristics of the included trials are shown in Table 1. A total of 625 patients were enrolled in the results synthesis. The GLP-1RAs evaluated in the studies included liraglutide (7 trials) and exenatide (3 trials). The follow-up time of most trials was approximately 6 months. The mean BMI was 29.4 kg/m^2^, and the mean HbA1c level was 8.1%. VAT and SAT were measured by computed tomography (CT) or magnetic resonance (MR). Nine trials reported the baseline values of VAT and SAT, with mean values of 188.1 cm^2^ for VAT and 274.5 cm^2^ for SAT [18–25, 27].

**Table 1:** Characteristics of the included studies.

Author	Year	Follow-up (weeks)	BMI		HbA1c (%)		Intervention	Number of patients	Instrument
			Mean	Range	Mean	Range			
Jendle [26]	2009	26	31.0	≤ 40	8.4	7.0–10.0	liraglutide 1.2 or 1.8 mg qd plus metformin vs.	68	CT
							glimepiride or placebo plus metformin	57	
Tanaka [19]	2015	24	28.5	≥ 23.5	7.6	6.9–9.4	liraglutide 0.9 mg qd plus metformin vs.	10	CT
							metformin	10	
Bouchi [25]	2016	36	28.0	≥ 25	8.0	7.0–10.0	liraglutide 0.9 mg qd plus insulin vs.	8	CT
							insulin	9	
Yan [23]	2019	26	29.8	20–35	7.7	6.5–10.0	liraglutide 1.8 mg qd vs.	24	MR
							sitagliptin or insulin glargine	51	
van Eyk [18]	2019	26	29.4	≥ 23	8.4	6.5–11.0	liraglutide 1.8 mg qd vs.	22	MR
							placebo	25	
Bizino [24]	2020	26	32.1	≥ 25	8.3	7.0–10.0	liraglutide 1.8 mg qd vs.	23	MR
							placebo	26	
Guo [22]	2020	26	28.7	> 25	7.4	> 6.5	liraglutide 1.8 mg qd vs.	31	MR
							insulin glargine or placebo	60	
Dutour [21]	2016	26	36.1	≥ 30	7.5	6.5–10.0	exenatide 10 μg bid vs.	22	CT
							oral antidiabetic therapy	22	
Liu [20]	2020	24	28.2	> 24	8.5	7.0–10.0	exenatide 10 μg bid vs.	38	MR
							insulin glargine	38	
Wang [27]	2020	26	23.7	18.5–25.0	8.5	7.0–10.0	exenatide 10 μg bid vs.	40	MR
							humalog mix25	41	

### 3.3 Risk of bias

Two trials did not provide the detailed process of random sequence generation (unclear risk) or the method of allocation concealment (unclear risk). Four trials did not mention whether the outcome assessment was blinded (unclear risk), and three trials were double-blind trials (low risk). Seven trials were supported financially by pharmaceutical manufacturers, and in accordance with the guidance provided by Cochrane, we assigned high risk to the item of “other bias” for these trials [28]. S1 Fig provides the quality assessment details.

### 3.4 Meta-analysis of outcomes by construct

**Fig 2:**
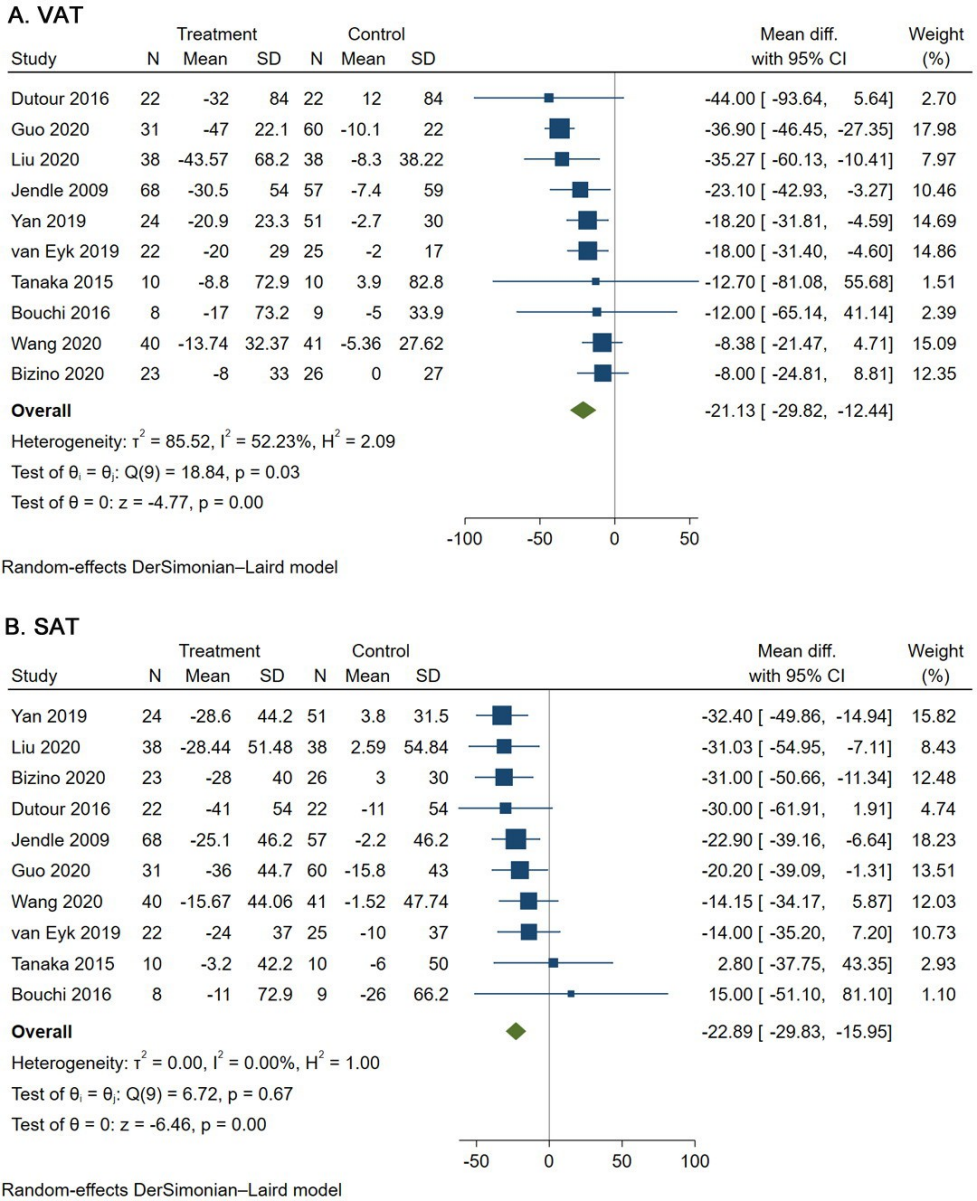
Random-effects pooled MD for VAT and SAT.

**Fig 3:**
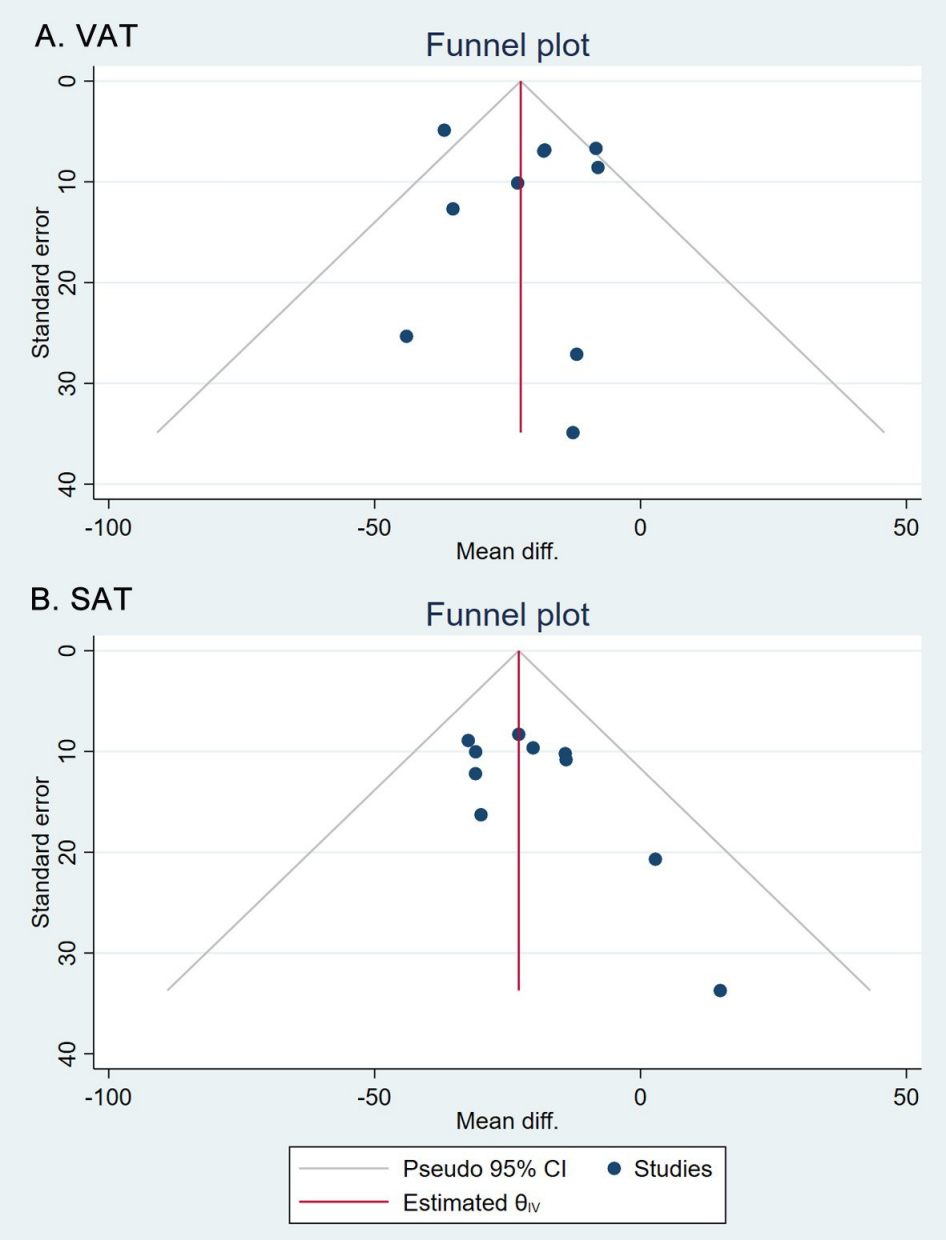
Begg’s funnel plots for publication bias of VAT and SAT.

Overall, compared to patients in the control groups, patients treated with GLP-1RAs showed significant reductions in VAT (MD -21.13 cm^2^, 95% CI [-29.82, -12.44]) and SAT (MD -22.89 cm^2^, 95% CI [-29.83, -15.95]) (Fig 2) [18–27]. We found substantial heterogeneity among studies for VAT (*I*^2^ = 52%, 95% CI [2%, 77%]) and less heterogeneity for SAT (*I*^2^ = 0%, 95% CI [0%, 62%]). No publication bias was found in the analysis of VAT (p = 0.910) and SAT (p = 0.211) by Egger’s test with funnel plots, as shown in Fig 3. As references, patients treated with GLP-1RAs also showed significant reductions in both body weight (MD -3.83 kg, 95% CI [-4.76, -2.89]) and HbA1c level (MD -0.38%, 95% CI [-0.66, -0.09]) compared with patients in the control groups (S2 Fig) [18–27].

Considering the wide confidence intervals for heterogeneity, sensitivity and subgroup analyses were further performed (Table 2). In the sensitivity analyses, GLP-1RA treatment was associated with a significant reduction in both VAT and SAT when only the studies that were published in 2019 and 2020 or the studies that directly provided SDs for the changes in adipose tissues were enrolled. Subgroup analyses were performed according to the type of GLP-1RAs, the baseline BMI and HbA1c level, and the MD in body weight and HbA1c level. GLP-1RA treatment was again associated with significant reductions in both VAT and SAT in all these subgroup analyses. Moreover, compared to the subgroup of MD in body weight < 3 kg, in the subgroup of MD in body weight ≥ 3 kg, GLP-1RA treatment resulted in more reductions in both VAT (MD -25.20 cm^2^, 95% CI [-36.38, -14.02] vs. MD -13.06 cm^2^, 95% CI [-22.27, -3.85]) and SAT (MD -23.95 cm^2^, 95% CI [-32.36, -15.53] vs. MD -17.63 cm^2^, 95% CI [-34.75, -0.50]).

**Table 2:** Sensitivity and subgroup analyses.

Study characteristics	Study number	Patient number	VAT (cm^2^)		SAT (cm^2^)	
			MD (95% Cl)	*I*^2^ (95% Cl)	MD (95% Cl)	*I*^2^ (95% Cl)
Published in 2019 and 2020	6	419	- 20.49 (- 31.45, - 9.53)	72% (35%, 88%)	- 24.03 (- 32.16, - 15.90)	0% (0%, 75%)
Provided SDs for the change in adipose tissue	8	561	- 20.58 (- 29.84, - 11.32)	61% (16%, 82%)	- 23.34 (- 30.57, - 16.12)	0% (0%, 68%)
Liraglutide	7	424	- 21.50 (- 31.21, - 11.79)	51% (0%, 79%)	- 22.93 (- 30.96, - 14.90)	0% (0%, 71%)
Exenatide	3	201	- 23.33 (- 46.27, - 0.04)	59% (0%, 88%)	- 23.33 (- 46.27, - 0.40)	59% (0%, 88%)
BMI baseline mean value ≥ 29 kg/m^2^	5	340	- 17.38 (- 24.95, - 9.81)	0% (0%, 79%)	- 25.96 (- 34.78, - 17.14)	0% (0%, 79%)
BMI baseline mean value < 29 kg/m^2^	5	285	- 24.27 (- 41.80, - 6.75)	69% (20%, 88%)	- 17.89 (- 29.15, - 6.63)	0% (0%, 79%)
HbA1c baseline mean value ≥ 8%	6	395	- 15.31(- 22.52, - 8.10)	1% (0%, 75%)	- 21.74 (- 30.49, - 12.99)	0% (0%, 75%)
HbA1c baseline mean value < 8%	4	230	- 29.03(- 42.82, - 15.23)	44% (0%, 81%)	- 24.85 (- 36.26, - 13.43)	0% (0%, 85%)
Mean difference in body weight ≥ 3 kg*	6	432	- 25.20(- 36.38, - 14.02)	58% (0%, 83%)	- 23.95 (- 32.36, - 15.53)	0% (0%, 75%)
Mean difference in body weight < 3 kg*	4	193	- 13.06(- 22.27, - 3.85)	0% (0%, 85%)	- 17.63 (- 34.75, - 0.50)	34% (0%, 77%)
Mean difference in HbA1c value ≥ 0.35%*	5	314	- 21.08(- 37.34, - 4.81)	76% (42%, 90%)	- 22.61 (- 32.68, - 12.54)	0% (0%, 79%)
Mean difference in HbA1c value < 0.35%*	5	311	- 19.66(- 28.07, - 11.25)	0% (0%, 79%)	- 23.15 (- 32.74, - 13.56)	0% (0%, 79%)

*, the mean difference indicates extra reduction in the GLP-1RA group compared to the control group.

Meta-regression was performed to evaluate the correlation between the MD in adipose tissue and the MD in body weight and HbA1c level. The MD in VAT showed a significant correlation with the MD in body weight (r = 6.324, p = 0.035) but no significant correlation with the MD in HbA1c level. The MD in SAT showed a significant correlation with neither the MD in body weight nor the MD in HbA1c level. The details of the meta-regression are shown in S3 Fig.

## 4. Discussion

This systematic review of 10 trials and 625 participants provided the first evidence that GLP-1RA treatment can significantly reduce VAT and SAT in patients with type 2 diabetes. When VAT and SAT baseline values were taken into consideration, GLP-1RA treatment seemed to be associated with the preferential reduction in VAT (11.23%) over SAT (8.34%). Moreover, GLP-1RA treatment resulted in a greater reduction in VAT and SAT in the subgroup with a greater reduction in body weight. In addition, the reduction in VAT was significantly correlated with the reduction in body weight.

Although both SAT and VAT are correlated with metabolic risk factors, VAT is more pathogenic than SAT [5, 29–31]. In the study of Karlsson et al with 325,153 patients, VAT was associated with an increased risk of hypertension, heart attack/angina, type 2 diabetes and hyperlipidaemia, and Mendelian randomisation analysis showed VAT to be a causal risk factor for all of the above four diseases [30]. VAT accumulation has also been linked with heart failure and cardiovascular death [32–35]. In early type 2 diabetes mellitus, VAT volume is associated with premature atherosclerosis independent of traditional risk factors [36]. A high ratio of VAT-to-SAT is a determinant of atherosclerosis and predicts cardiovascular events in patients with type 2 diabetes [37, 38]. Therefore, there is no doubt that VAT is an emerging risk factor for type 2 diabetes, atherosclerosis, and cardiovascular disease [39].

The recognition of increased VAT as a cardiac risk factor has increased interest in strategies that target these adipose tissues. Several systematic reviews have confirmed that strategies including exercise, calorie restriction and pharmaceutical interventions can reduce both VAT and SAT [40–42]. Notably, the absolute reduction in the VAT area has been shown to be similar to that in the SAT area with exercise (-26.3 cm^2^ vs. -31.5 cm^2^), whereas the reduction in the VAT area has been shown to be approximately half that of the SAT area with calorie restriction (-33.6 cm^2^ vs. -65.1 cm^2^) [40]. Moreover, exercise interventions have been shown to result in a greater reduction in VAT relative to weight loss than pharmacological interventions [41].

The abnormal distribution of VAT and SAT is very common in patients with type 2 diabetes. After adjusting for age, sex, BMI and waist circumference, the patients with type 2 diabetes had a higher VAT area (163.79 ± 47.98 cm^2^) than the patients without diabetes (147.49 ± 39.09 cm^2^) [43]. Therefore, it is important to identify an antidiabetic drug that can reduce VAT while decreasing glucose.

The present study demonstrated that GLP-1RA treatment can significantly reduce VAT in patients with type 2 diabetes. Since the patients in the study of Nobarani et al had a BMI similar to that in our meta-analysis (30.7 kg/m^2^ vs. 30.0 kg/m^2^), we can take this study as a reference [43]. The absolute reduction in VAT by GLP-1RAs (21.13 cm^2^) in the present study was larger than the difference between patients with and without type 2 diabetes in the reference (16.3 cm^2^) [43]. Therefore, we believe that this extent of reduction in VAT by GLP-1RA treatment should have great clinical significance. Moreover, GLP-1RA treatment led to similar reductions in VAT and SAT, and this feature is similar to that of exercise but different from that of calorie restriction.

Whether the VAT reduction induced by GLP-1RA treatment is due to a specific modulation or is the result of overall weight loss is unknown. GLP-1RA treatment can decrease appetite and calorie intake, and a hypocaloric diet can lead to reductions in VAT [40–42]. The meta-regression of the present research also demonstrated that the mean reduction in VAT was significantly correlated with the mean reduction in body weight. Therefore, the contribution of weight loss to the reduction in VAT induced by GLP-1RAs cannot be ruled out.

However, some evidence indicates that GLP-1RA treatment may have distinct effects on VAT and SAT reduction. Real-time PCR and immunofluorescence results have shown that the GLP-1 receptor is present and more abundant in VAT and EAT than in SAT [11]. In an animal study, liraglutide redistributed body fat by decreasing VAT and relatively increasing SAT, which could partly be attributed to changes in the expression of the corresponding key enzymes for lipid metabolism. Lipogenesis is reduced in visceral white adipose tissue but is elevated in subcutaneous white adipose tissue [44]. In rodents, the activation of central GLP-1 receptors contributes substantially to an increase in sympathetic outflow, and the central action of GLP-1RAs might induce specific lipolysis in VAT compared to SAT [45, 46]. In the present meta-analysis, GLP-1RA treatment showed similar absolute and higher percentage reduction in VAT compared to SAT, suggesting that GLP-1Ras may activate additional pathways that calorie restriction does not.

There are some limitations in our research. First, although all these included studies were RCTs, more than half were not double blind, and the majority of trials were supported by pharmaceutical manufacturers, which leads to more favourable efficacy results and conclusions than sponsorship by other sources [28]. Second, the number of studies was relatively large, but the total number of patients was small. Third, significant heterogeneities were detected in the analyses of VAT, which may be due to differing demographic characteristics and large differences in the intervention type and design. Moreover, two studies did not directly provide the SD for the changes in adipose tissues [19, 21]. Therefore, sensitivity and subgroup analyses were performed, and GLP-1RA treatment still showed significant reductions in both VAT and SAT. Fourth, the number of studies included in the meta-regression was fairly small, which may lead to result instability. Finally, unlike previous meta-analyses that included patients who were overweight and/or obese, the present research focused on patients with type 2 diabetes, and these patients had high levels of VAT. Therefore, whether GLP-1RA treatment has similar effects as exercise in VAT reduction in patients with type 2 diabetes needs to be further confirmed by well-designed and directly comparable trials.

In conclusion, GLP-1RA treatment led to significant and similar absolute reductions in VAT and SAT. The reduction in adipose tissues with GLP-1RA treatment may be correlated with the reduction in body weight. In combination with the fact that excessive VAT is associated with a higher risk of cardiovascular diseases and mortality, the findings of the present study support the evidence for a protective role of GLP-1RAs in cardiovascular disease.

## Supporting information

S1 Fig Cochrane risk of bias assessment. (TIF)

S2 Fig Random-effects pooled weight MD for HbA1c level and body weight. (TIF)

S3 Fig The correlation between the MD in adipose tissue (VAT and SAT) and the MD in body weight and HbA1c level. VAT, visceral adipose tissue; SAT, subcutaneous adipose tissue.
(TIF)

S1 Table PRISMA checklist. (DOCX)
