# Supplementary figures and images for: The effects of glucagon-like peptide-1 receptor agonists on adipose tissues in patients with type 2 diabetes: A meta-analysis of randomised controlled trials
Source: PLoS One. 2022 Jul 7;17(7):e0270899. doi: 10.1371/journal.pone.0270899 (PMC9262225; doi:10.1371/journal.pone.0270899)

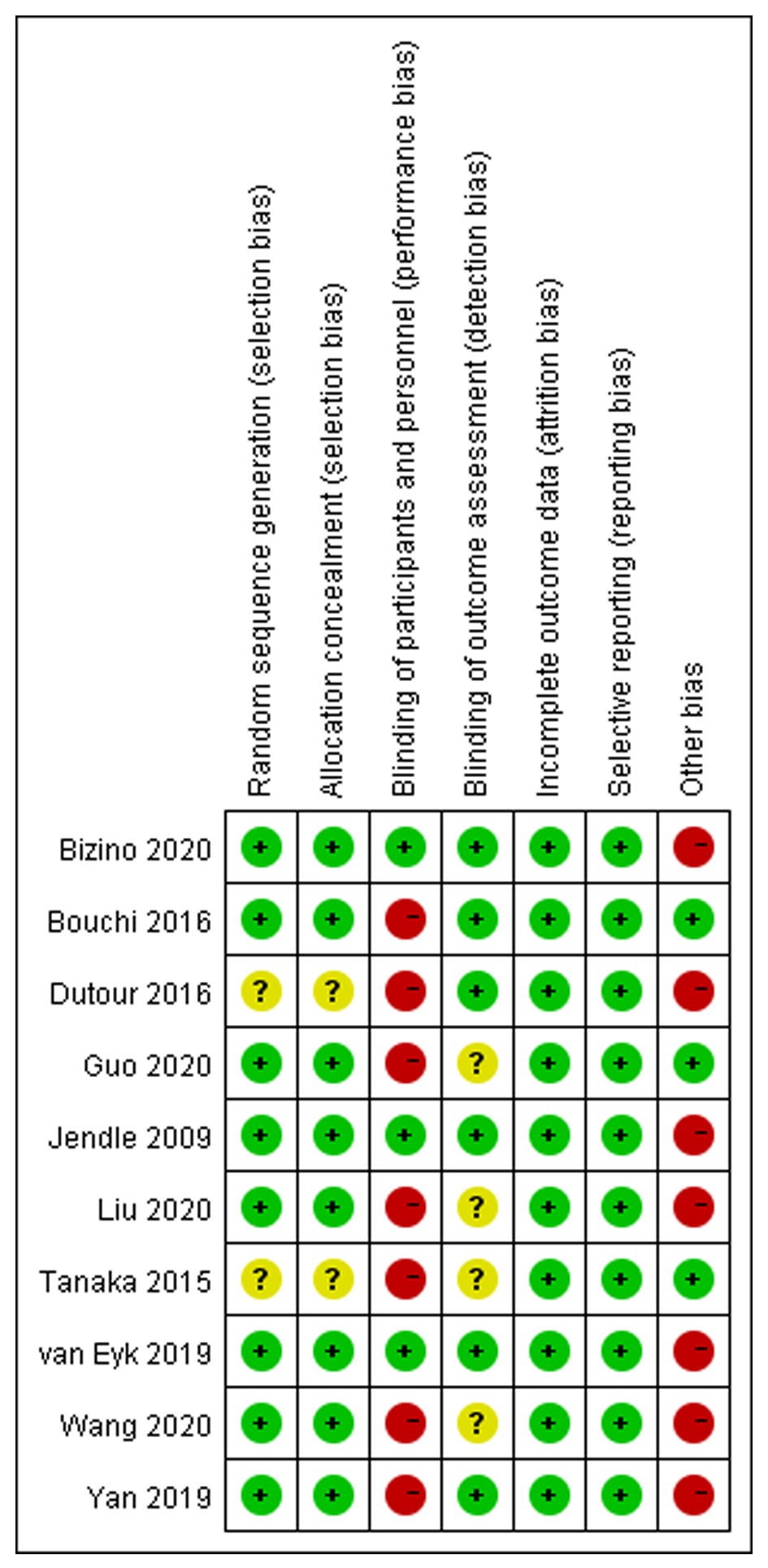

Supplement: S1 Fig — (TIF) [file pone.0270899.s001.tif]

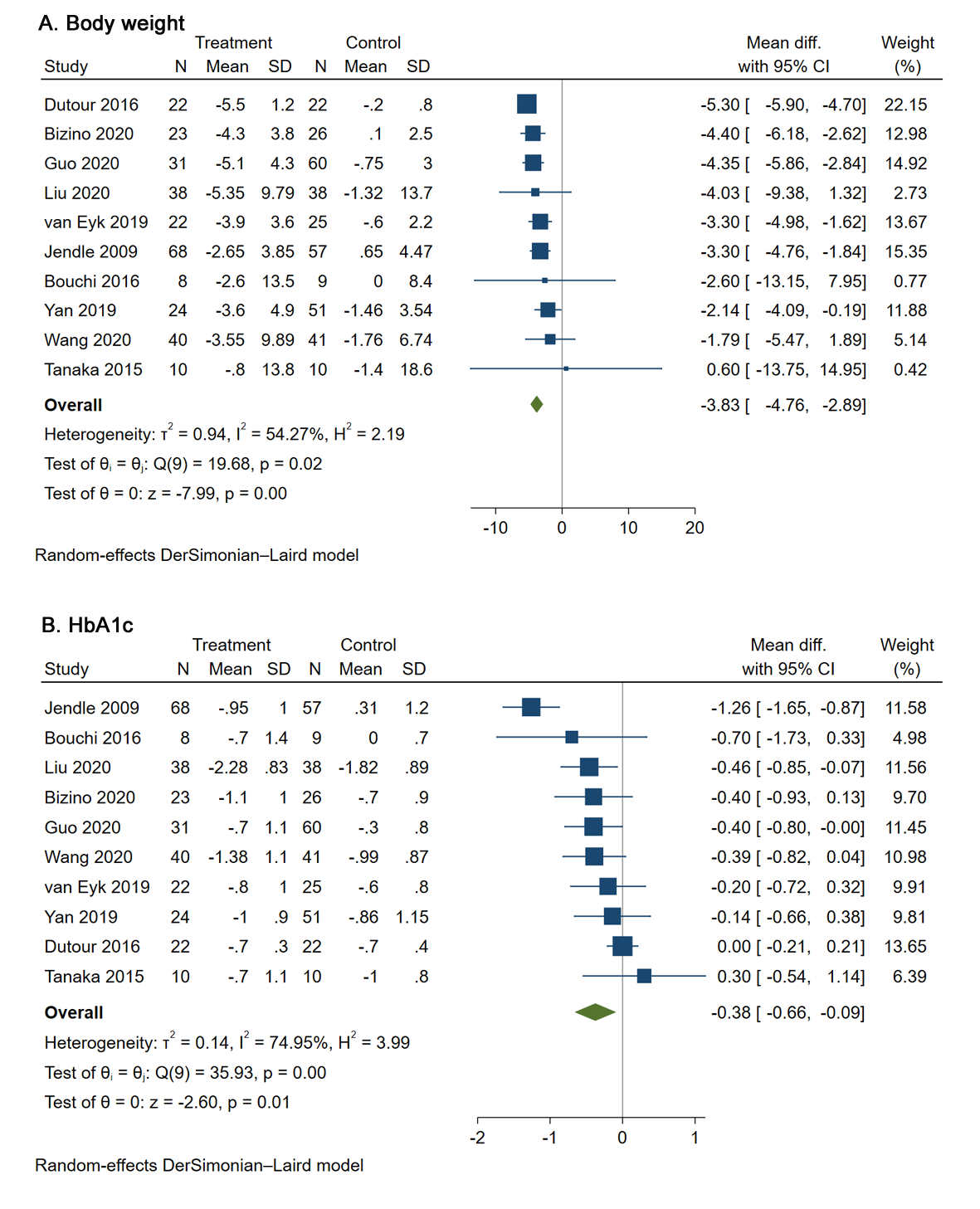

Supplement: S2 Fig — (TIF) [file pone.0270899.s002.tif]

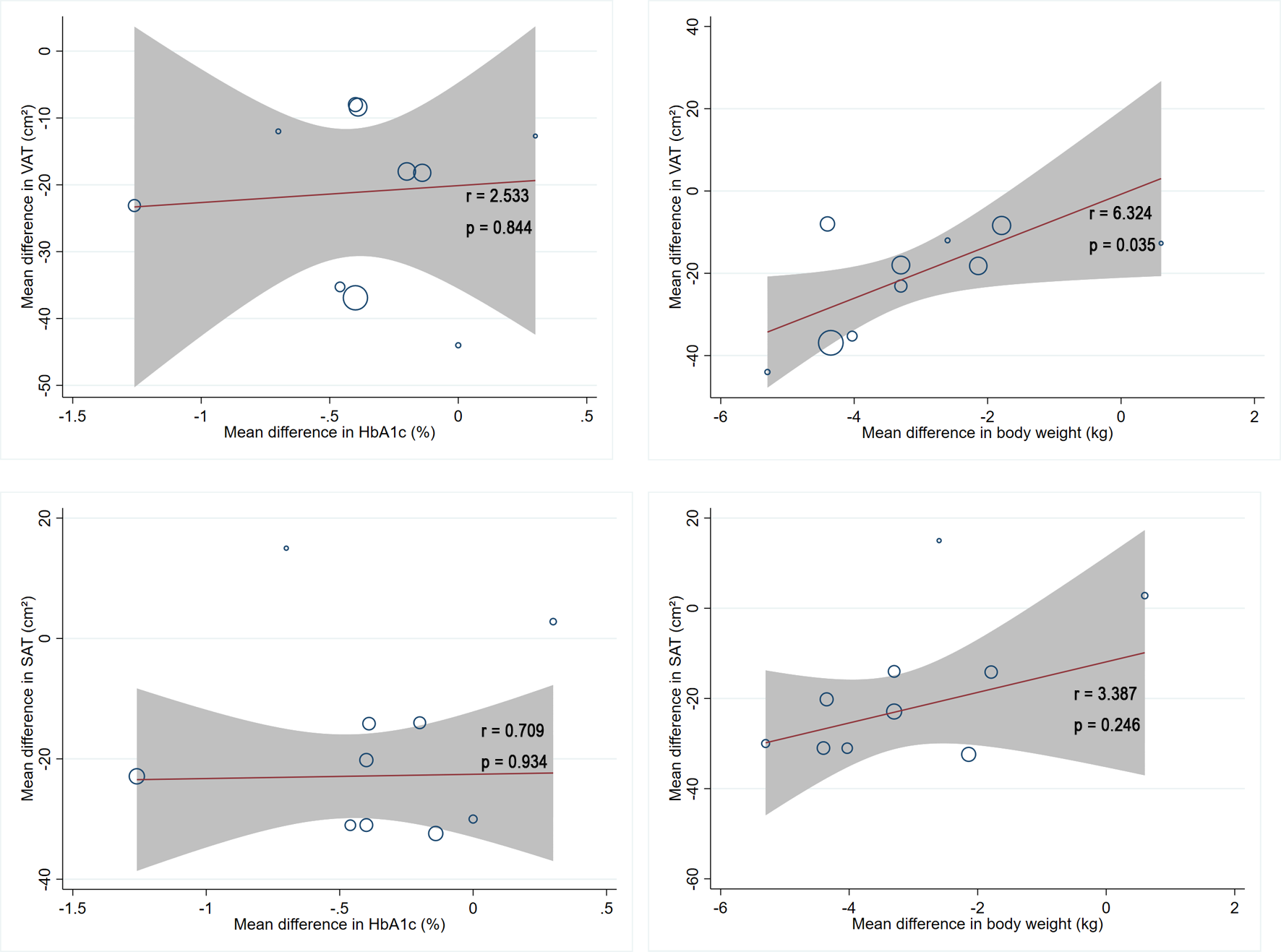

Supplement: S3 Fig — VAT, visceral adipose tissue; SAT, subcutaneous adipose tissue. (TIF) [file pone.0270899.s003.tif]
